# Surface configuration of microarc oxidized Ti with regionally loaded chitosan hydrogel containing ciprofloxacin for improving biological performance

**DOI:** 10.1016/j.mtbio.2022.100380

**Published:** 2022-08-08

**Authors:** Rui Zhou, Ying Zhou, Jiahui Cheng, Jianyun Cao, Ming Li, Hailing Yu, Daqing Wei, Baoqiang Li, Yaming Wang, Yu Zhou

**Affiliations:** a State Key Laboratory for Mechanical Behavior of Materials, Xi'an Jiaotong University, Xi'an, 710049, PR China; b The Second Affiliated Hospital of Xi'an Jiaotong University (Xibei Hospital), Xi'an, 710004, PR China; c Key Laboratory of LCR Materials and Devices of Yunnan Province, School of Materials and Energy, Yunnan University, Kunming, 650500, PR China; d Honghui Hospital, Xi'an Jiaotong University, Xi'an, 710054, PR China; e The Fifth Affiliated Hospital, Sun Yat-Sen University, Zhuhai, Guangdong Province, 519000, PR China; f Department of Materials Science and Engineering, Harbin Institute of Technology, Harbin, 150080, PR China

**Keywords:** Microarc oxidation coating, Cchitosan hydrogel, Cciprofloxacin, Rregionally loaded structure, Bbioactivity, Aantibacterial ability

## Abstract

The bacterial colonization and poor osseointegration of Ti implants significantly compromise their applications in load-bearing bone repair and replacement. To endorse the Ti with both excellent bioactivity and antibacterial ability, we developed a microarc oxidation coating that was modified uniformly by hydroxyapatite (HA) nanodots arrays and loaded regionally with chitosan hydrogel containing ciprofloxacin. The bonding between the HA nanodots covered coating and the chitosan hydrogel is further enhanced via silanization and chemical grafting of glutaraldehyde. Benefiting from the regionally loaded structure of the chitosan hydrogel, the chitosan hydrogel unloaded area can promote the cell adhesion and proliferation with excellent bioactivity, though relatively low OD value of cck8 has been observed at the beginning of the cell culturing. Whereas, the OD value of cck8 rises with the prolongation of the cell culturing time due to the degradation of the regionally loaded chitosan hydrogel. With the help of the laden ciprofloxacin in chitosan hydrogels, the sample effectively sterilizes the bacterial with a bacteriostatic ring. Therefore, regional loading of chitosan hydrogel containing ciprofloxacin on the modified microarc oxidation coating is a good approach to endorse Ti with both excellent bioactivity and antibacterial ability.

## Graphical abstract

Benefiting from the regionally loaded chitosan hydrogel with ciprofloxacin, the surface configuraed Ti exhibits excellent antibacterial ability, apatite-inducing ability and cell activity.

**Figure:**
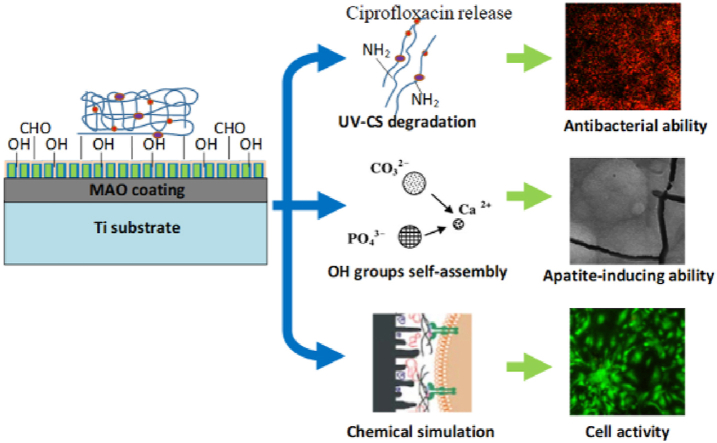

## 1 Introduction

Ti is a promising biomaterial with good biocompatibility, high strength, and excellent toughness, and thus is widely used as implant for tooth roots or joints [1]. However, the bio-inertness and bacterial colonization of Ti limit their wide application in clinical treatment [2]. The surface of implant greatly affects its bioactivity and antibacterial ability, which plays a vital role in regulating the interaction between tissues and implants during the healing [3,4]. Recently, great efforts have been focused on the surface modification of Ti to obtain a safe, non-toxic and antibacterial implant [5].

Microarc oxidation (MAO) is a useful strategy to enhance the bioactivity of Ti [6]. The porous surface structure of MAO coating exhibits high surface energy, which can promote albumin and cell adhesion [7,8]. Meanwhile, the dopping of the metal ions into the MAO coating can not only improve its bioactivity but also endorse it with antibacterial ability [9]. As a traditionally inorganic antimicrobials, the Zn doped MAO coating can suppress the growth of bacteria with a maximum antibacterial rate of 76.8% [9]. However, this antibacterial rate is too low to meet the requirements for application [10].

To achieve excellent antibacterial performance for medical implanted devices, Yang and et al. fabricates smart antibacterial coatings through the layer-by-layer assembly, which exhibits tunable antibiotics release behaviors and efficient antibacterial activities [[11], [12], [13]]. Rajendran et al. prepare a gel coating containing vancomycin on Ti surface, which not only improves the cell activity but also effectively inhibites the growth of staphylococcus aureus via the release of vancomycin [14]. Noticeably, chitosan is non-toxic and bioactive molecules with broad-spectrum antibacterial ability [15]. The spin-coated chitosan hydrogel on the surface of anodic oxidized Ti can inhibit the adhesion of bacteria and form a bioactive thin film [16]. Interestingly, the modified chitosan with OH groups can be easily processed into the water based solution, promoting the killing of bacterial via changing their physiological function based on adsorption [17]. In addition, a UV-crosslinked hydrogel with designed structure also can be fabricated in the bassis of the modified chitosan, which can be applied as a drug delivery system to sterilize the bacterial [18]. Noticeably, ciprofloxacin not only exhibits good activity against bacterials for disinfection, but also can stimulate the activity of bone marrow mesenchymal stem cells with a reasonable dose for bone repair [19]. Thus, fabricating a ciprofloxacin loaded chitosan hydrogel coating on Ti would be a good approach to enhance its antibacterial performance.

Although the drug loaded chitosan hydrogel coating can endow Ti with excellent antibacterial ability, it hinders the bonding between implants and tissues during bone repair [20]. Particularly, a gap would be left at the interface after the degradation of the hydrogel [21], leading to failure of the surgery. To reduce the size of the gap caused by the hydrogel degradation, regional loading of chitosan hydrogel containing ciprofloxacin on the surface of microarc oxidized Ti would be a potential method to simultaneously achieve good bioactivity and antibacterial ability.

Herein, the conventional MAO coating on Ti was hydrothermally treated to form a layer of bioactive HA nanodots [22]. Then, chitosan hydrogel containing ciprofloxacin was regionally loaded on the HA nanodots covered MAO coating surface to impart the Ti with antibacterial ability. To ensure good bonding between the chitosan hydrogel and the modified MAO coating, alkoxysilane and aldehyde were chemically grafted onto the coating surface to form the chemical bonding. Due to the changing of surface functional groups after the treatment of silanization and chemical grafting of aldehyde, it also provides good platform to study the effect of surface functional groups on the bioactivity and antibacterial ability of the Ti.

## 2 Materials and methods

### 2.1 Materials preparation

The pure titanium was cut into circle plate with the size of φ14.7 ​mm ​× ​1 ​mm. Then, 240 #, 600 # and 1000 # sandpapers were successively used to grind the plates until there was no obvious scratch on the surface. Anhydrous ethanol was used to clean the sample surface for 10 ​min in an ultrasonic cleaner to remove any contaminant. Then, the sample was cleaned with deionized water and dried for further experiments. The prepared pure titanium plates were treated by MAO power supply (Liquid phase plasma 20K). The pure aluminum plate was connected to the anode of MAO power supply, and the stainless-steel plate was used as the cathode. The electrolyte for MAO contains EDTA-2Na (15 ​g/L), calcium dihydrogen phosphate (12 ​g/L), and NaOH (5 ​g/L). A set of processing parameters was used for MAO, including applied voltage (350 ​V), frequency (1 ​kHz), duty ratio (10%), and applied time (5 ​min). To form HA nanodots, the MAO coatings were hydrothermally treated in a NaOH solution (pH of 12.5) at 100 ​°C for 16 ​h. The chemical grafting was performed on the hydrothermally treated samples to improve the adhesion between hydrogel and coating. The detailed process is described as bellow: after soaking in 3-aminopropyltriethoxysilane (98%) for 24 ​h, the samples were annealed at 110 ​°C for 2 ​h for silanization; then they were further immersed in 5% glutaraldehyde solution for 3.5 ​h to realize dehydration synthesis with the glutaraldehyde on the surface. As for the preparation of hydrogel, chitosan acetic acid solution (2 w/v%) was grafted with photo-crosslinking group by adding methacrylic anhydride at 60 ​°C for 24 ​h. The modified chitosan was obtained after dialysis and lyophilization. To fabricate the regionally loaded chitosan hydrogel, a PDMS template was prepared according to the designed structure (600 ​μm ​× ​600 ​μm ​× ​500 ​μm with a intergap of 1500 ​μm). Then the modified chitosan solution (25 ​mg/mL) was loaded on sample surface accroding to the template structure via ultraviolet light irradiating for 8 ​min. Demoulding of the hydrogel was performed after freeze-drying. In the following, the samples were labbeled as Ti-MAO, HT, CG and RLCH accroding to the surface treatment as shown in Table 1. Meanwhile, the chitosan is represented as CS, the modified chitosan is represented as mCS, and the chitosan hydrogel after UV cross-linking is represented as UV-CS.

**Table 1:** Sample code based on the surface treatment.

Sample code	Microarc oxidation	Surface treatment		
		Hydrothermal treatment	Chemical grafting	Regional loading chitosan hydrogel
Ti-MAO	✓			
HT	✓	✓		
CG	✓	✓	✓	
RLCH	✓	✓	✓	✓

**Table 2:** Minimum inhibitory concentration (MIC) of the antibacterial agents loaded on the samples.

Samples	Antibacterial agent	Minimum inhibitory concentration of ciprofloxacin	
		E. Coli	*S. aureusS. aureus*
Ciprofloxacin	Ciprofloxacin	0.01∼0.1 ​μg/mL	0.1∼1 ​μg/mL
Ciprofoxacin loaded UV-CS	Ciprofloxacin and m-CS	0.001∼0.01 ​μg/mL	0.01∼0.1 ​μg/mL

### 2.2 Structure characterization

A scanning electron microscope (SEM, Hitachi SU6600, Japan) was used to observe the surface morphologies of the samples with different surface features. The phase compositions of the samples were analyzed by X-ray diffraction (XRD, D/max-gB, Japan). Transmission electron microscope (TEM, Tecnai G2F30, USA) and X-ray photoelectron spectroscopy (XPS, K-Alpha, USA) were used to observe the structures and chemical states of the samples before and after chemical grafting. Laser confocal microscope (CLSM, Olympus3000, Japan) was used to investigate the surface morphology and roughness of the samples. The degradation of the as-prepared hydrogel was tested via calculating the weight after immersing in the PBS for different time. Meanwhile, nuclear magnetic resonance spectroscopy (NMR, AVANCE III HD 600 ​MHz, Switzerland) and fourier transform infrared spectroscopy (FTIR, Magna-IR 560 ​E ​.S.P., USA) were used to characterize the chemical structure of the as-prepared hydrogel.

### 2.3 Biological performance characterization

The samples with different surface features were immersed in simulated body fluid (SBF) for 1 day to test their apatite-inducing ability. *Escherichia coliEscherichia coli* (*E. Coli*) and staphylococcus aureus (*S. aureus*) were used to study the antibacterial ability of the samples. The bacteria have been cultured in agar medium to the fourth generation before testing. The solution containing 1 ​× ​x10^5^ ​CFU/mL bacterial was dropwise added to the 24-well plate with samples. The plate was cultivated in a constant temperature incubator for 24h. Then, the bacterial liquid was aspirated, and the samples were washed with 0.9% NaCl solution. The A and B solutions for live and dead staining were added to the wells respectively. After incubating for 15 ​min minutes at room temperature, the samples were washed with 0.9% NaCl solution. The stained morphology of the samples was observed under a fluorescence microscope. As for the bacteriostatic ring testing, bacterial solution was evenly smeared on the petri dish with agar medium. Then, the sterilized samples were placed in the middle of the petri dish, which was incubated in a constant temperature incubator at 37 ​°C for 24 ​h. The growth of bacteria around the sample was observed. To quantitatively evaluate the antibacterial performance of the material, minimum inhibitory concentration (MIC) of the ciprofloxacin and the UV-CS containing ciprofloxacin are measured by macro-broth dilution method.

The bioactivity of the samples with different surface features was tested through cell experiments using human bone marrow mesenchymal stem cells (hBMSCs). The incubated cell solution was added to the 24-well plate where the samples were placed, then the cells were cultured in a cell incubator for one day and seven days, respectively. Cell viability was assessed by cck8. The number of live cells attached to the surface was calculated via live and dead stained image.

## 3 Results and discussion

**Fig. 1:**
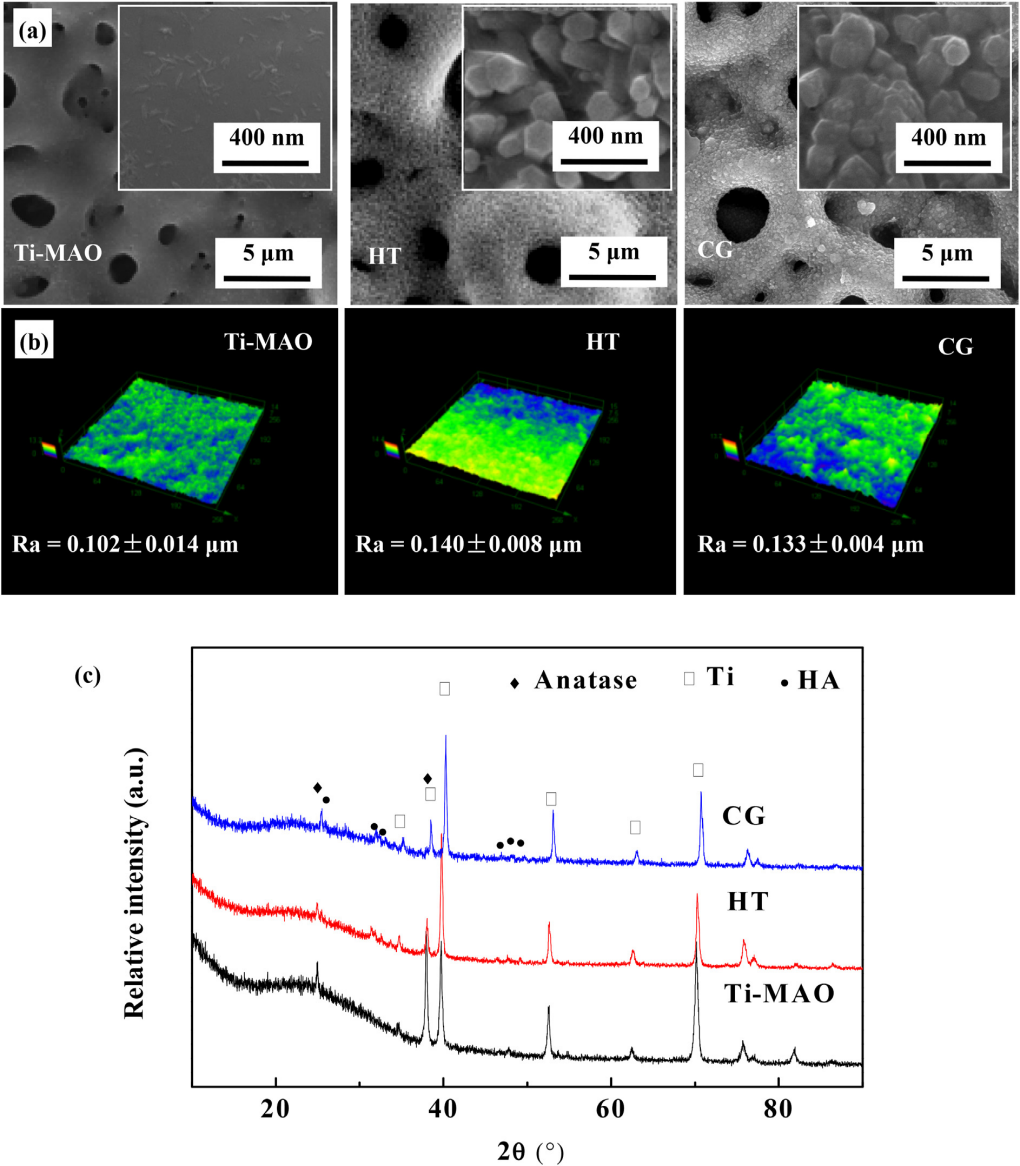
Surface structure and XRD spectra of the coatings with different features: (a) SEM morphology, (b) laser confocal morphology, roughness, and (c) XRD spectra of the Ti-MAO, HT and CG.

The surface morphologies and XRD patterns of the samples with different surface features are shown in Fig. 1(a) and (c). Clearly, the MAO coating shows a typical micro scale porous structure. As expected, a nanodot arrays have been formed on the sample surface after the hydrothermal treatment. Combined with the XRD results, it is clear that the MAO coating consists of anatase phase TiO_2_, while the as-formed nanodot arrays on the hydrothermally treated surface is made of HA.

As the behavior of cells is greatly affected by the adhered surface structure, the laser confocal microscopehas been used to investigate the surface morphology and roughness of the samples before and after chemical grafting (Fig. 1(b)). Clearly, the arithmetic average roughness (Ra) of the sample increased after hydrothermal treatment due to the formation of the HA nanodots, but there is no obvious difference in both the morphology and the roughness between the HT and CG samples. This phenomenon indicates that chemical grafting does not change the surface structure of the hydrothermally treated sample, but would greatly change the surface functional group [23].

**Fig. 2:**
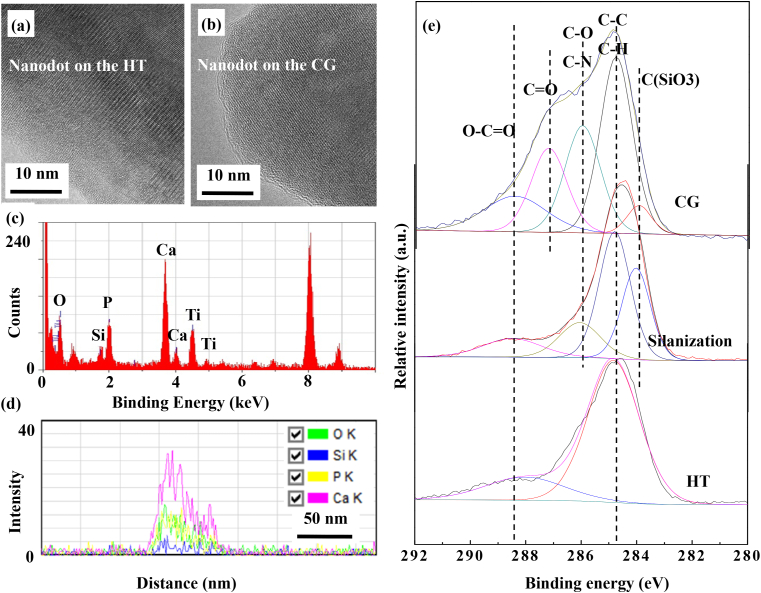
TEM and XPS analysis for the HT surface before and after chemical grafting: (a) HRTEM image of the nanodot on the HT, (b) HRTEM image of the nanodot on the CG, (c) EDS spectrum of the nanodot on the CG, (d) EDS line scanning image of the nanodot on CG along the radial direction, and (e) high resolution XPS spectra of C detected from the HT surface before and after chemical grafting.

To further confirm the states of functional groups on sample surface before and after chemical grafting, high resolution transmission electron microscope (HRTEM) and XPS have been used to study the structure, elemental distribution and chemical states of the samples. Fig. 2(a) and (b) are the HRTEM images of the sample before and after chemical grafting. Associated considering the EDS results (Fig. 2(c) and (d)), it is obvious that several layers of atoms enriched with Si have been deposited on the HA nanodot surface after the grafting of the functional groups via salinization and chemical grafting of glutaraldehyde [24]. Briefly, the silanol group of the silane molecule can join with the hydroxyl group on the HT surface [25,26]. While, the amino group at the other end of the silane molecule can react with an aldehyde group of glutaraldehyde [23]. The aldehyde group of glutaraldehyde could crosslink with the amino group of the chitosan hydrogel [27], which is expected to enhance the bonding strength between the coating and the loaded chitosan hydrogel. As expected, the C–-Si–-O and C=N groups have been observed on the CG surface (verified by the C 1s spectra shown in Fig. 2(e)); at the same time, the C=O of aldehyde group has also been observed on the surface of CG sample that have been treated in the glutaraldehyde solution (Fig. 2(e)) [28].

**Fig. 3:**
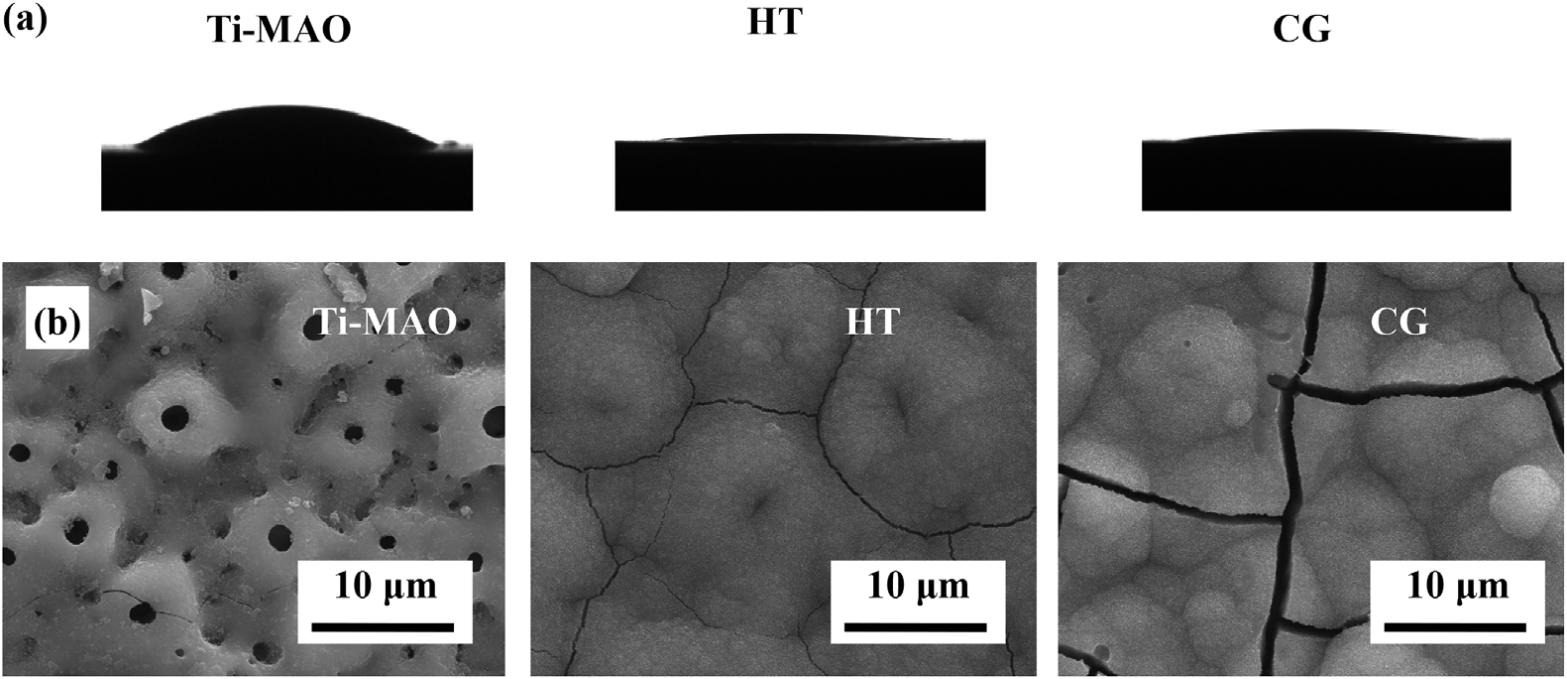
Contact angle and apatite-inducing ability of the samples with different surface features: (a) contact angle images of the Ti-MAO, HT and CG, and (b) the surface morphologies of the Ti-MAO, HT and CG after SBF immersing for 1 day.

**Fig. 4:**
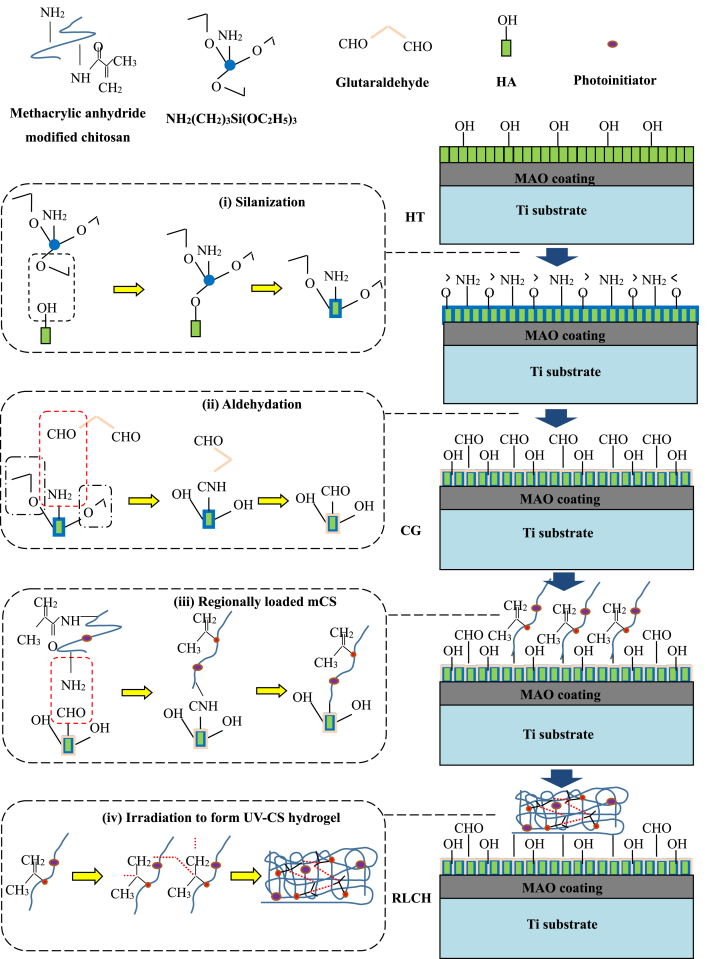
Schematic diagram of the chemical bonding between the UV-CS hydrogel and modified coating surface via chemical grafting.

It should be noted that the surface functional group can greatly affect the hydrophilicity of the surface (Fig. 3(a)), which is a dominating factor in the bioactivity of the materials [29]. As expected, the water contact angle decreases from 35°º to 3°º after the hydrothermal treatment. To ensure a good hydrophilicity of the modified surface, a relatively low concentration of glutaraldehyde has been used for the chemical grafting together with a short reaction time, as the replace of surface functional group from Si–-OH to C=O would reduce the surface hydrophilicity [30,31]. In addition, the side reaction of aldol condensation would also greatly reduce the number of hydroxyl groups on the surface. To evaluate the bioactivity of the surface after chemical grafting, the samples with different surface features have been immersed in simulated body fluid (SBF) for 1 day to test their apatite-inducing ability (Fig. S1). Compared with the HT sample, the apatite inducing ability of the sample after chemical grafting would be weakened, as only gel film has been formed on the surface of the one treated with glutaraldehyde for long soaking time (Fig. S1). As reported previously, the hydroxyl groups are formed on the surface after hydrothermal treatment, which can promote the nucleation and growth of apatite [32]. After silanization, the as-formed silanol groups have similar functions to titanium hydroxyl groups and also exhibit apatite-inducing ability (Fig. 4) [33]. Besides, the chemical grafting of aldehyde groups that do not have apatite inducing ability would reduce the bioactivity of the CG samples (Fig. 4(ii)) [34]. Therefore, the bioactivity of the CG can be controlled in a certain range by adjusting the concentration of glutaraldehyde during the chemical grafting process (Fig. S1). As shown in Fig. 3 (b), the CG sample treated with the glutaraldehyde solution at a concentration of 5% for 3.5 ​h can effectively induce the formation of apatite on the surface after only one day in the SBF, exhibiting good bioactivity.

**Fig. 5:**
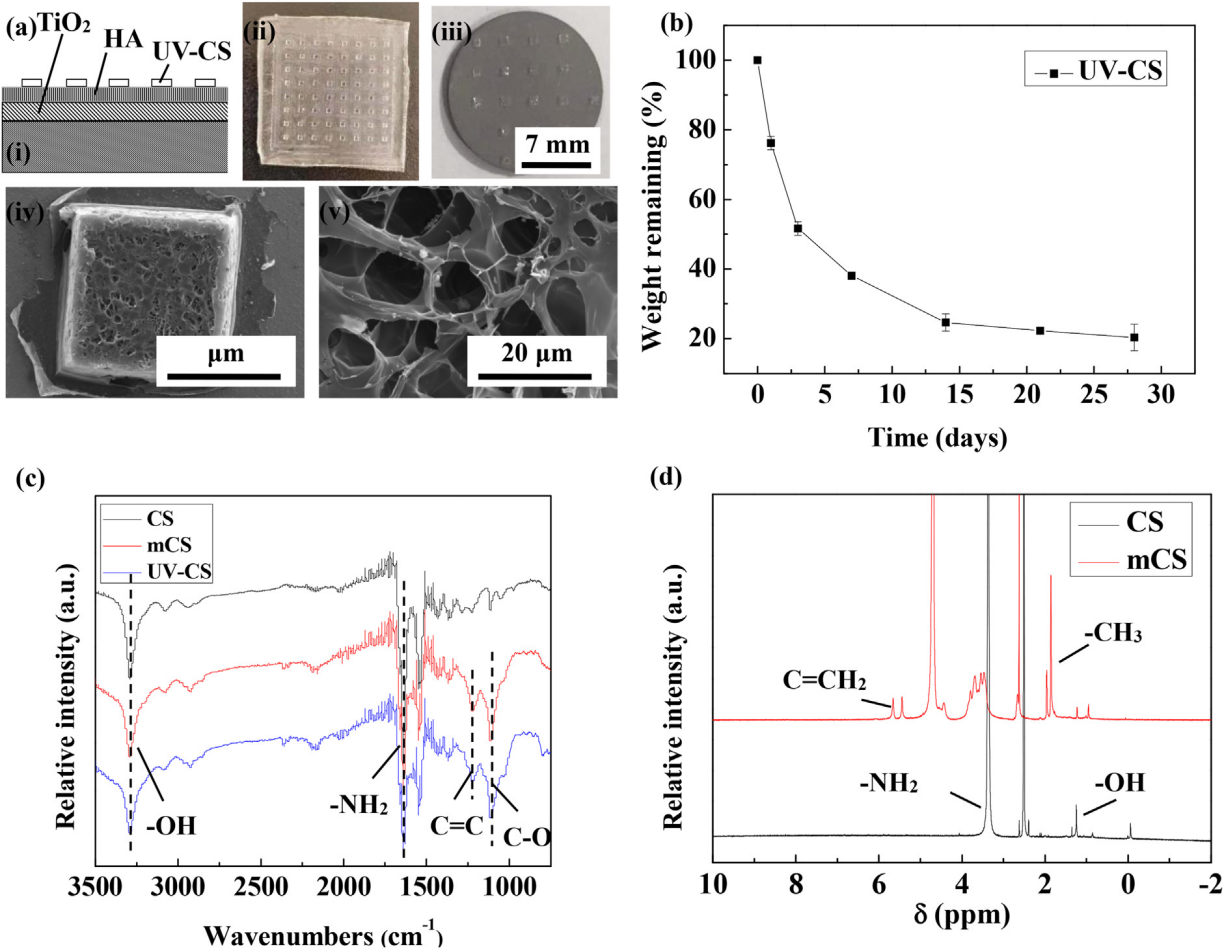
Schematic diagram and ananlysis for the chemical grafted sample with regionally loaded chitosan hydrogel (RLCH): (a) the fabrication and SEM image of the regionally loaded hydrogel on the RLCH after freeze drying: (i) schematic diagram for the structure of the RLCH, (ii) image of PDMS model, (iii) image of the lyophilized RLCH, (iv) SEM morphology of the lyophilized chitosan hydrogel, and (v) magnified SEM image of the lyophilized chitosan hydrogel, (b) degradation performance of the UV-CS soaking in PBS, c) the FTIR spectra of the CS, mCS and UV-CS, and (d) 1H NMR spectra of the CS and mCS.

It is expected to endow the sample with antibacterial ability via loading chitosan hydrogel on the MAO coating, but the bioactivity of the sample would be significantly reduced before the degradation of the chitosan hydrogel [35]. Inspired from the micromechanical technology, regional loading of antibacterial chitosan hydrogel on the sample surface after chemical grafting would be a good approach to maintain the bioactivity of the sample before the total degradation of chitosan hydrogel. With the help of UV irradiation, the C=C bond on the modified chitosan chain is activated for polymerization, and then the chitosan hydrogel with network chain structure is formed on the sample surface according to the PDMS mask (Fig. 4 (iv)). In terms of the unloaded area, the sample maintains its original appearance (Fig. 5(a) (iii)). As for the loaded area, the lyophilized hydrogel shows a porous network structure (Fig. 5(a) (v)).

After implanting into the body, the regionally loaded chitosan hydrogel on the RLCH sample would quickly degrade into the micro-enviorment. The degradation of the hydrogel not only leads to the release of antibacterial chitosan but also the exposure of the bioactive surface. For this reason, it is necessary to test the degradability of chitosan hydrogel. As shown in Fig. 5 (b), the hydrogel rapidly degraded at the initial stage of soaking PBS solution, and basically degraded after 4 weeks immersion. Benefiting from this degradability of the chitosan hydrogel, the RLCH is expected to be an advanced material with both excellent bioactivity and antibacterial ability [36].

To ensure the UV crosslink ability of the modified chitosan with methacrylic anhydride and the chemical grafting between the sample surface and the chitosan hydrogel, the chemical structure of the modified chitosan and hydrogel are characterized. A strong absorption band of the stretching vibration of hydroxyl group at 3340 ​cm^−-1^ appears in the FT-IR spectra of CS, mCS and UV-CS samples (Fig. 5(c)). The band at 3000-3300 ​cm^−-1^ points to the C–-H stretching vibration of C–-H at unsaturated C. Meanwhile, the bending vibration of the amino group of the chitosan at 1600 ​cm^−-1^ is also observed. All these characteristic bands belong to the functional groups on chitosan. Different from chitosan, new absorption bands at 1430 ​cm^−-1^ and 1050 ​cm^−-1^ have been observed from the spectra of the mCS and UV-CS, which belong to the C=C and C–-O bonds on methacrylic anhydride. It means that methacrylic anhydride has been successfully grafted onto the chitosan molecular chain. As well known, methacrylic anhydride is a UV-crosslinking group that can polymerize under UV light irradiation [27]. Therefore, by grafting methacrylic anhydride onto the molecular chain of chitosan, a UV cross-linkable modified chitosan hydrogel is allowed to the regionally loaded on the chemical grafted sample surface.

To further confirm the chemical structure of the modified chitosan, the 1H NMR spectra of CS and mCS have been analyzed (Fig. 5(d)). The peaks at 2.5 ​ppm and 4.7 ​ppm are corresponding to the solvent peaks of DMSO and D_2_O. As for the chitosan, the peak at 1.3 ​ppm is corresponding to the hydroxyl peak of chitosan, while the peak at 3.5 ​ppm points to the amino group of chitosan. In terms of the modified chitosan, new absorption peaks at 1.9 ​ppm and 5.5 ​ppm are obtained, which are the methyl proton peaks and olefin proton peaks on methacrylic anhydride. As adequate dialysis has been conducted to the modified chitosan solution before freeze-drying, no small molecules could remain in it. Therefore, all these results confirm the successful grafting of methacrylic groups to the chitosan molecular chain, which plays the important role of the patterning for the UV cross-linked hydrogel. Besides, no obvious change in amino group among the chiston, mCS and UV-CS has been observed. This phenomenon confirms the chemical bonding between the UV cross-linked hydrogel and the chemical grafted surface via the intermediate of glutaraldehyde as shown in Fig. 4.

**Fig. 6:**
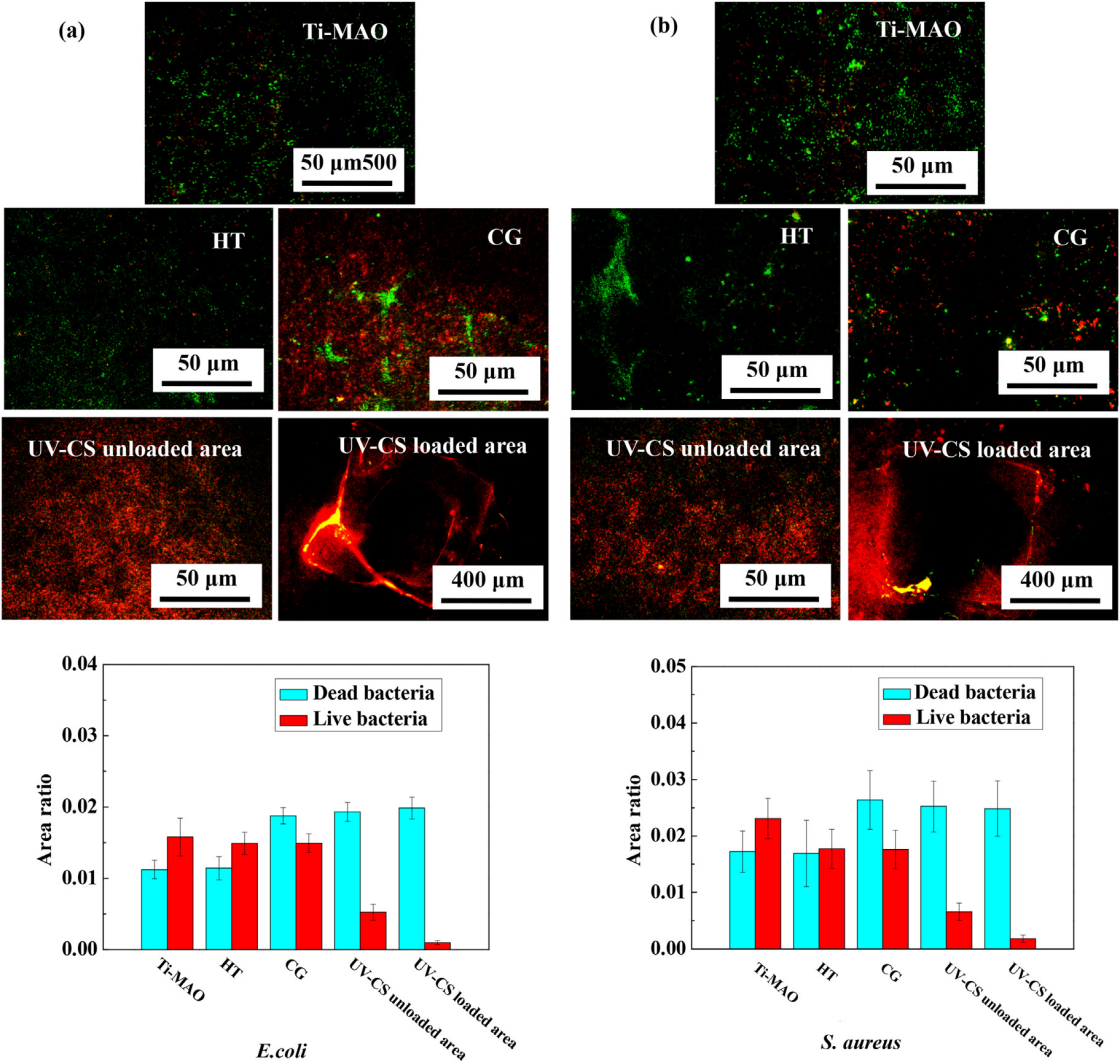
Live (green) / dead (red) staining images together with quantiﬁed bacteria numbers incubated on the samples with different surface features for 1 day: (a) *E. Coli* group, and (b) *S. aureus* group.

To test the broad-spectrum antibacterial ability of the sample, *E. Coli* and *S. aureus* are respectively incubated on the samples with different surface features for 24 ​h. The live and dead staining morphometric analysis results are shown in Fig. 6. As for the *E. Coli* experimental group, obvious difference in the stained morphology has been obtained. Clearly, the surface of the Ti-MAO is almost covered by live bacteria, because the basic MAO coating does not have antibacterial ability. The number of attached bacteria decreases slightly after chemical grafting compared with that of the HT sample, this phenomenon is likely due to the grafting of the antibacterial glutaraldehyde on the sample surface. Noticeably, a large number of dead bacteria has been observed in the UV-CS unloaded area on RLCH, showing good antibacterial ability. In terms of the UV-CS loaded area, live bacteria are rarely observed. The bright red colour appears at the border of the hydrogel is due to the adsorption of the dye. Normally, chitosan is insoluble in water, which greatly limits its antibacterial ability in the body. Herein, the good antibacterial ability of the RLCH samples even at the UV-CS unloaded area is due to the degradation of the UV-CS. As the modified chitosan molecules are water-soluble, it enables to be hydrolyzed during the antibacterial process, widely releasing to efficiently kill the *E. Coli*. The antibacterial rates are 66.94% and 93.90% (compared with Ti-MAO) for the UV-CS unloaded and loaded areas, respectively.

As for the *S. aureus* experimental group, similar experimental results have been obtained. The RLCH sample still shows better antibacterial ability than other samples, its antibacterial rate is 71.52% and 92.12% (compared with Ti-MAO) for the UV-CS unloaded area and loaded area respectively.

Consistent with the live/dead stained images, the adhesion and proliferation of the bacteria is significantly limited by the regionally loaded UV-CS, only a small number of bacteria can be observed from the UV-CS unloaded area (Fig. S2). However, the bacteria shows slightly difference in proliferation behavior, which should be caused by the different antibacterial mechanisms of chitosan for gram-negative bacteria and gram-positive bacteria [37]. Normally, the macromolecular chitosan is adsorbed on the surface of the *E. coli* (gram-negative bacteria), which forms a polymer film to prevent the transportation of nutrients, causing the death of bacteria [38]. While, newly divided bacteria without polymer film has been observed in the UV-CS unloaded area (Fig. S2(a)). It means that the water soluble m-CS in this work is hard to deposit on the surface of *E. coli*, leading to a relatively low antibacterial rate in the UV-CS unloaded area (66.94% when compared with Ti-MAO). As for the gram-positive bacteria like *S. aureus*, the antibacterial mechanism is that the positive charge of chitosan interacts with the negative charge at the surface of bacteria, leading to the loss of the normal physiological function of the bacteria [39]. Herein, the water soluble m-CS can interacts with the surface of *S. aureus* as expected, thus only several one with small size has been observed in the Fig. S2(b). Therefore, the RLCH can exhibit good broad-spectrum antibacterial ability thanks to the degradation of the regionally loaded UV-CS.

**Fig. 7:**
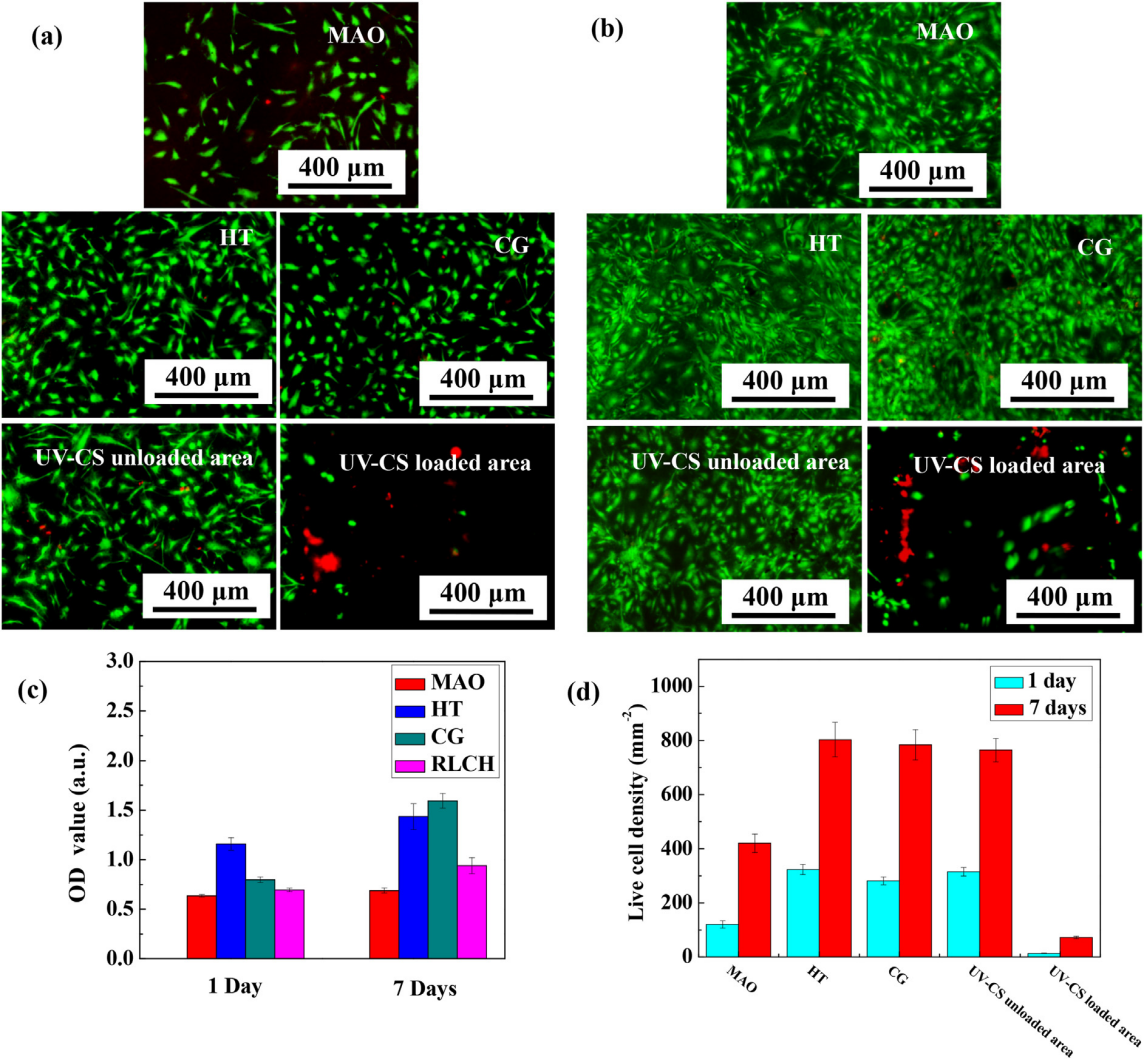
Adhesion and proliferation of the hBMSCs on the on the samples with difference surface features: live (green) / dead (red) staining images of the hBMSCs after being incubated on the samples for (a) 1 day and (b) 7 days, (c) metabolic activities, and (d) the quantiﬁed cell numbers of hBMSCs incubated on the samples.

Although the regionally loaded chitosan hydrogel on the chemical grafted surface can impart good antibacterial ability to the sample, the loaded chitosan hydrogel may also be toxic to cells while killing bacteria. To clarify the effect of UV-CS on cells, the cell viability after culturing for 1 day and 7 days have been studied (Fig. 7). After culturing for 1 day, the number of hBMSCs on the HT and CG samples is higher than that of the Ti-MAO, showing obvious bioactivity. As for the RLCH, clear differences in live/dead stained images have been obtained in the two different areas. The number of live cells in the UV-CS loaded area is very small, while the number of those in the UV-CS unloaded area is similar to that of the CG surface. The reason for this is because of the degradation of chitosan hydrogel. Clearly, the chitosan hydrogel rapidly degrades as mCS again in the first 3 days (Fig. 5(b)) in PBS. Because the released mCS is water soluble, the cells which lose attachment to the degraded chitosan hydrogel are easy to fall off from the UV-CS surface. Therefore, a small number of cells has been observed from the image in the UV-CS loaded area after culturing for 1 day, though it has been reported that chitosan film can promote the attachment and growth of osteoblasts [40]. Similar results have been observed after culturing for 7 days, the number of live cells attached on the samples increases, especially in the UV-CS loaded area on RLCH. The significant rise of cell number in the UV-CS loaded area is also related to the degradation of chitosan hydrogel. Firstly, less mCS would release into the liquid medium after culturing for 7 days, therefore the microenvironment is more suitable for cell when compared with that at the early stage of the hydrogel degradation. Secondly, the degradation rate for the remained chitosan hydrogel is greatly reduced as shown in Fig. 5(b). It means that the remaining chitosan hydrogels become relatively stable for cell attachment and proliferation. Thus, the number of cells desorbed from the hydrogel surface caused by degradation is reduced. All these reasons lead to a gradual increasing in the number of adhered cells on the surface of the UV-CS loaded area after culturing for 7 days. And as time goes by, the loaded UV-CS would be completely degraded, then the bioactive coating would expose again and show excellent bioactivity as that of the HT sample. Hence, it is clear that the relative low OD value and live cell number on the RLCH is attributed to the degradation of the hydrogel, but not the toxicity. In addition, chitosan hydrogel has been reported as a biocompatible material due to the biological specific interaction between its functional groups and the cell membrane receptor [41]. Therefore, regional loading of chitosan hydrogel on the surface hydrothermally treated MAO coating can impart both good biocompatibility and bioactivity to the Ti implants.

**Fig. 8:**
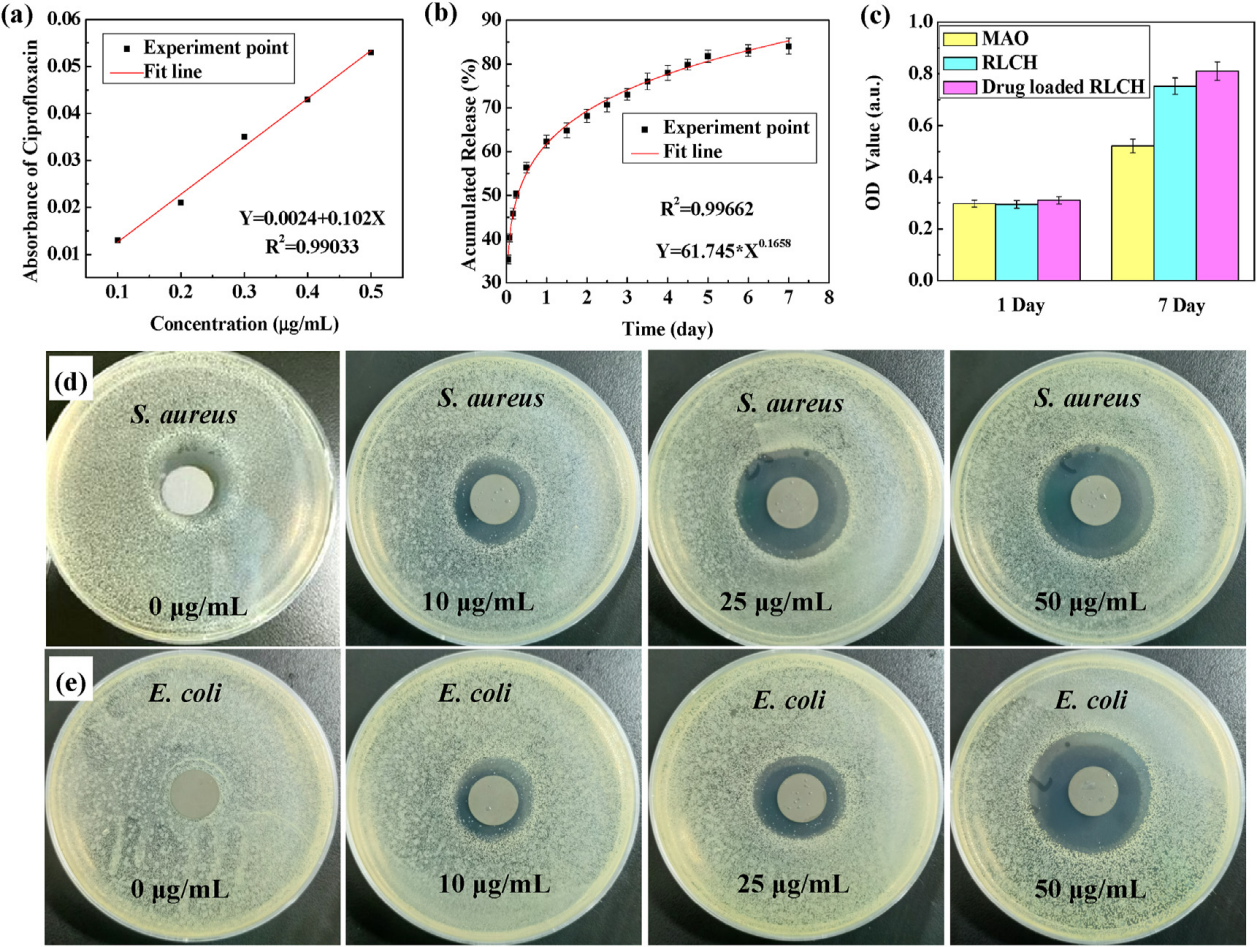
The biological perfermance of ciprofloxacin loaded RLCH: (a) the standard release curve of ciprofloxacin, (b) the release kinetics of ciprofloxacin from UV-CS hydrogel after a regular interval of time at 37 ​°C, (c) metabolic activities of the hBMSCs incubated on the ciprofloxacin loaded RLCH with the concentration of 25 ​μg/mL, (d) bacteriostatic ring images of the ciprofloxacin loaded RLCH with different concentrations for *S. aureus*, and (e) bacteriostatic ring images of the ciprofloxacin loaded RLCH with different concentrations for *E. Coli*.

To meet the requirements of high antibacterial rate in clinical application, the regionally loaded chitosan hydrogel on RLCH has been used as drug delivery system for ciprofloxacin. Fig. 8(a) is the standard curve of ciprofloxacin in PBS, which reveals a good linear correlation between drug concentration and absorbance of ciprofloxacin (R^2^ ​= ​0.99033). Fig. 8(b) is the release kinetics of ciprofloxacin from UV-CS hydrogel after a regular interval of time at 37 ​°C. The *in vitro* diffusion data are fitted according to the Korsmeyer-Peppas equation (Y=*K*∗X^*n*^). The correlation coefficient (R^2^) is 0.99662, indicating a good correlation between the experimental data and the Korsmeyer-Peppas model. From the data fitting, the transport constants (*K*) and transport exponents (*n*) were 61.745 and 0.1658 respectively. It means that the release kinetics model for ciprofloxacin from chitosan hydrogel belongs to fickian diffusion model, revealing a weak interaction between hydrogel skeleton and ciprofloxacin. Meanwhile, Fig. 8(b) also shows that the ciprofloxacin loaded RLCH obviously slows down the release performance for the drug with the help of UV-CS. Furthermore, recent works have reported that ciprofloxacin is a broad-spectrum antibiotic and does not exhibit adverse effect on BMSCs when applied with low dose [19,42]. Consistent with those reports, the ciprofloxacin loaded RLCH does not show any biological toxicity according to the cytotoxicity tests (Fig. 8(c)). Notably, the OD value of CCK8 for the ciprofloxacin loaded RLCH is slightly higher than those of the Ti-MAO and RLCH, suggesting ciprofloxacin could improve the activity of hBMSCs. Clear, the bacteriostatic ring test reveals that the RLCH can only sterilize the growth of *S. aureus*. Whereas the ciprofloxacin loaded RLCH with a dose of 10 ​μg/mL can effectively sterilize the growth of both *S. aureus* and *E. Coli*, forming an obvious bacteriostatic ring. Furthermore, the diameter of bacteriostatic ring becomes larger with the increase in loaded dose of ciprofloxacin, confirming the further enhancement in sterilizing ability (Fig. 8(d) and (e)). As for traditional oral antibiotics, the daily dose of ciprofloxacin is 6 ​mg/kg, while the effective drug concentration in tissue fluid is 5 ​μg/mL. In terms of the ciprofloxacin loaded RLCH with a dose of 10 ​μg/mL, the total amount of drug loaded on the surface is only 0.036 ​μg. Therefore, regional loading of chitosan hydrogel containing ciprofloxacin on the modified MAO coating can achieve effective antibacterial ability at low loadings of antibiotic, avoiding the side effects caused by the abuse of antibiotics.

To quantitatively evaluate the antibacterial performance of the material, minimum inhibitory concentration (MIC) of the ciprofloxacin and the UV-CS containing ciprofloxacin are measured by macro-broth dilution method (Table 2). Clearly, the *E. Coli* is intensitive to ciprofloxacin, exhibiting a low MIC of 0.01–∼0.1 ​μg/mL. In terms of the *S. aureus*, the MIC of ciprofloxacin is 0.1–∼1 ​μg/mL, which is higher than that of *E. Coli*. It should be noticed that the MIC of ciprofloxacin loaded on RLCH becomes smaller for both *E. Coli* and *S. aureus* than that of the pure ciprofloxacin, which is attributed to the synergistic antibacterial effects of m-CS and ciprofloxacin. In detail, as the carrier of ciprofloxacin, chitosan hydrogel significantly slows down the release performance for the drug, reducing the number of drug administration. Secondly, the loaded drug on Ti surface improves the utilization of ciprofloxacin in chitosan hydrogel, which can obtain a higher concentration than MIC in local area due to the existence of concentration gradient. Meanwhile, the adsorbed mCS changes the permeability of bacterial cell membrane [38], which disturbs the normal physiological activities of bacteria but does not affect the entry of ciprofloxacin. Thus, a relatively high concertation of ciprofloxacin is expected inside the bacteria, which can act on the subunit of DNA helicase [43]. The sufficient ciprofloxacin inhibits DNA synthesis and replication, leading to the death of bacterial. Therefore, the synergistic effect of ciprofloxacin and modified chitosan allows the ciprofloxacin loaded RLCH to effectively sterilize the growth of both *S. aureus* and *E. coli* with low loadings.

## 4 Conclusion

In summary, to promote the expression of both biological activity and antibacterial ability, chitosan hydrogel containing ciprofloxacin was regionally loaded on the hydrothermally treated MAO coating that was covered by HA nanodots arrays. By control of the surface functional groups during the chemical grafting process, the modified surface not only exhibits excellent apatite-inducing ability, but also benefits the bonding between chitosan hydrogel and MAO coating surface. Thanks to the degradation of chitosan hydrogel, the as-prepared sample can effectively inhibit the growth of bacteria on the sample surface. Benefiting from the regionally loaded structure of the chitosan hydrogel, the unloaded area can promote the cell adhesion and proliferation with excellent bioactivity at the beginning of the culturing. As for the chitosan hydrogel loaded area, cells can proliferate on the hydrogel with prolonged culturing time. Thanks to the synergistic effect of ciprofloxacin and modified chitosan on antibacterial performance, the ciprofloxacin loaded RLCH can effectively sterilize the growth of bacterial. Thus, the concept of surface configurated Ti with regionally loaded chitosan hydrogel containing ciprofloxacin would be a promising strategy for developing Ti implants with excellent biological performance.

## CRediT authorship contribution statement

**Rui Zhou:** Conceptualization, Methodology, Formal analysis, Investigation, Resources, Writing – original draft, Writing – review & editing, Funding acquisition. **Ying Zhou:** Methodology, Formal analysis, Investigation, Writing – original draft. **Jiahui Cheng:** Methodology, Formal analysis, Investigation, Validation. **Jianyun Cao:** Methodology, Investigation, Writing – review & editing. **Ming Li:** Methodology, Investigation, Resources, Funding acquisition, Writing – review & editing. **Hailing Yu:** Investigation, Resources. **Daqing Wei:** Methodology, Writing – review & editing, Funding acquisition. **Baoqiang Li:** Methodology, Writing – review & editing. **Yaming Wang:** Methodology, Writing – review & editing. **Yu Zhou:** Supervision, Project administration.

## Declaration of competing interest

The authors declare that they have no known competing financial interests or personal relationships that could have appeared to influence the work reported in this paper.
