# Supplementary material for: Surface configuration of microarc oxidized Ti with regionally loaded chitosan hydrogel containing ciprofloxacin for improving biological performance
Source: Mater Today Bio. 2022 Aug 8;16:100380. doi: 10.1016/j.mtbio.2022.100380 (PMC9399291; doi:10.1016/j.mtbio.2022.100380)
Supplement: Multimedia component 1 [file mmc1.docx]

**Supplementary material**

**Surface configuration of microarc oxidized Ti with regionally loaded chitosan hydrogel containing ciprofloxacin for improving biological performance**

Rui Zhou,*^a^ Ying Zhou,^a^ Jiahui Cheng,^b^ Jianyun Cao,^c^ Ming Li,^d^ Hailing Yu,^e^ Daqing Wei,^f^ Baoqiang Li,^f^ Yaming Wang ^f^ and Yu Zhou ^f^

a State Key Laboratory for Mechanical Behavior of Materials, Xi’an Jiaotong University, Xi’an 710049, P.R. China.

b The Second Affiliated Hospital of Xi’an Jiaotong University (Xibei Hospital), Xi'an, 710004, P.R. China.

c Key Laboratory of LCR Materials and Devices of Yunnan Province, School of Materials and Energy, Yunnan University, Kunming 650500, P.R. China.

d Honghui Hospital, Xi’an Jiaotong University College of Medicine, Xi’an 710054, P.R. China.

e The Fifth Affiliated Hospital, Sun Yat-Sen University, Zhuhai, Guangdong Province 519000, P. R. China.

f Department of Materials Science and Engineering, Harbin Institute of Technology, Harbin, 150080, P. R. China.

**Corresponding Author**

*Rui Zhou. E-mail: [yxhzr@163.com](mailto:yxhzr@163.com)


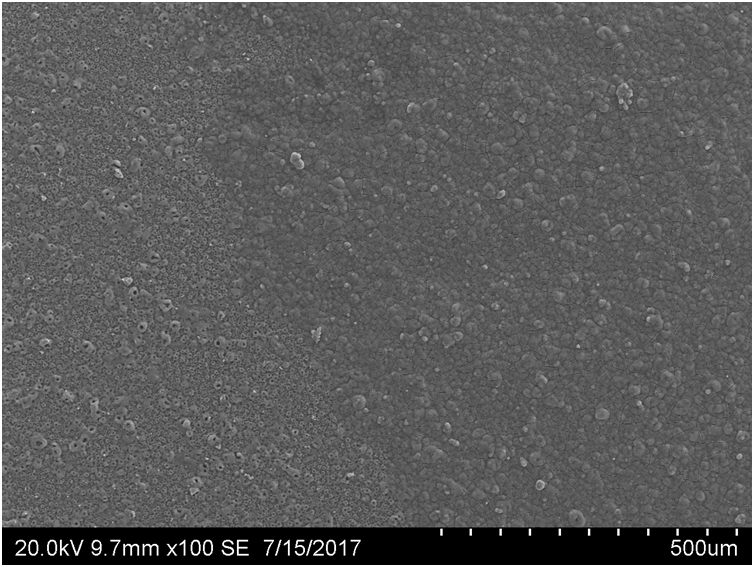

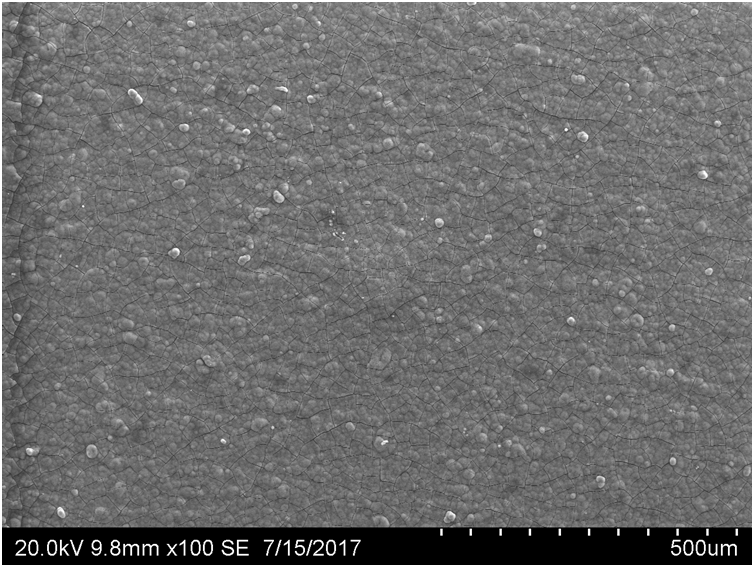

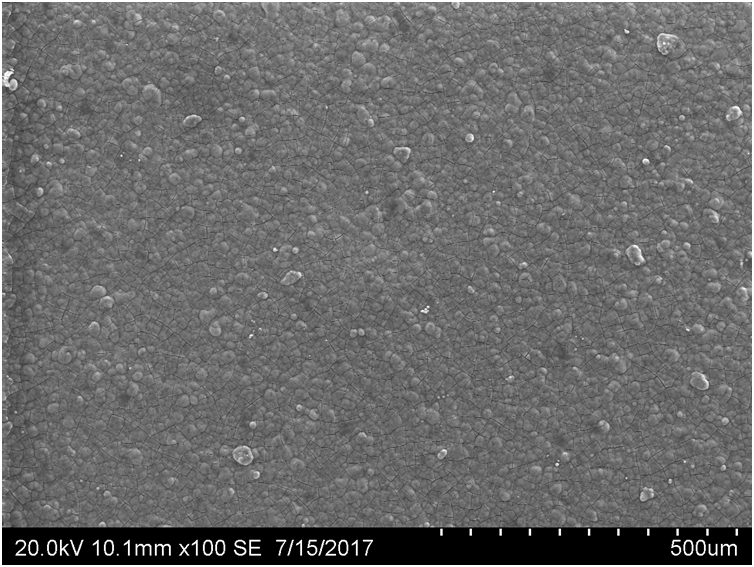

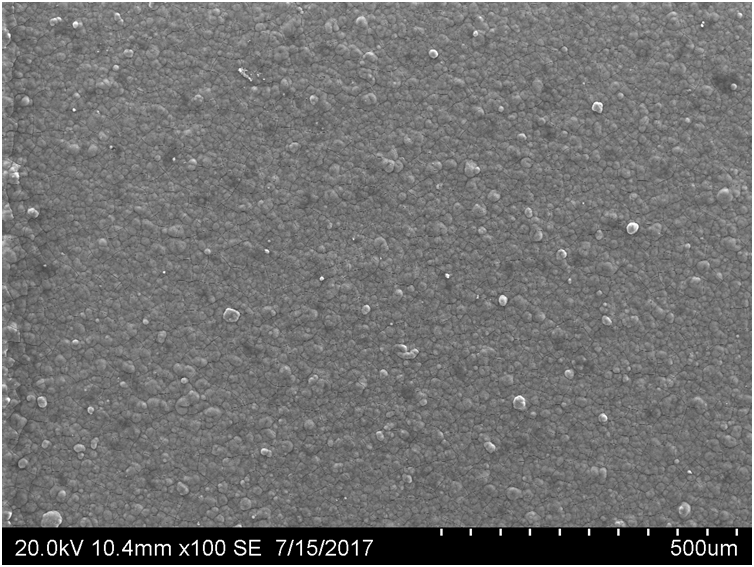


**100 μm**

**(a)**


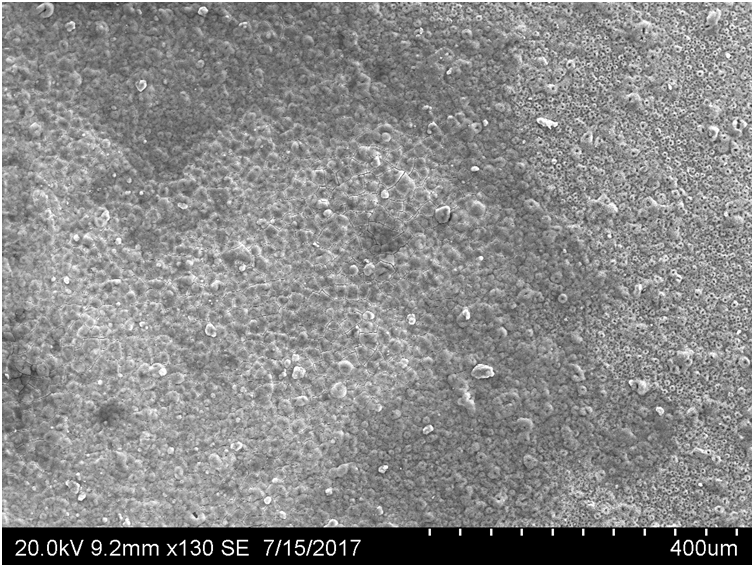

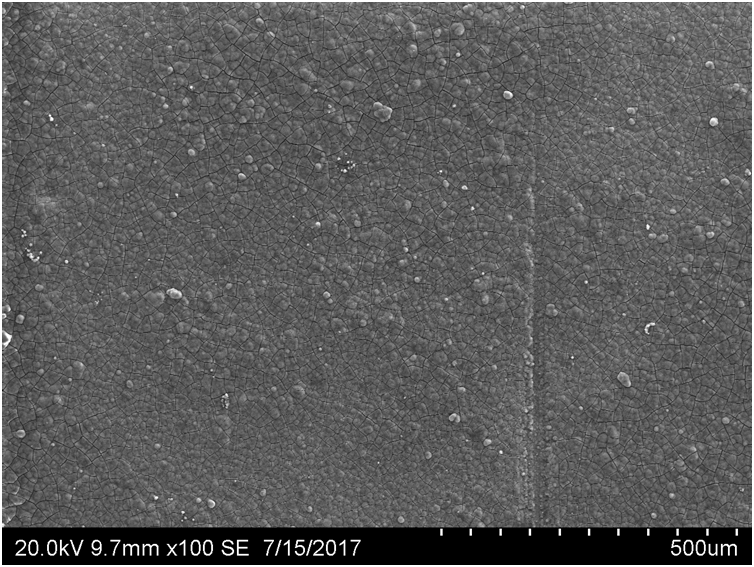

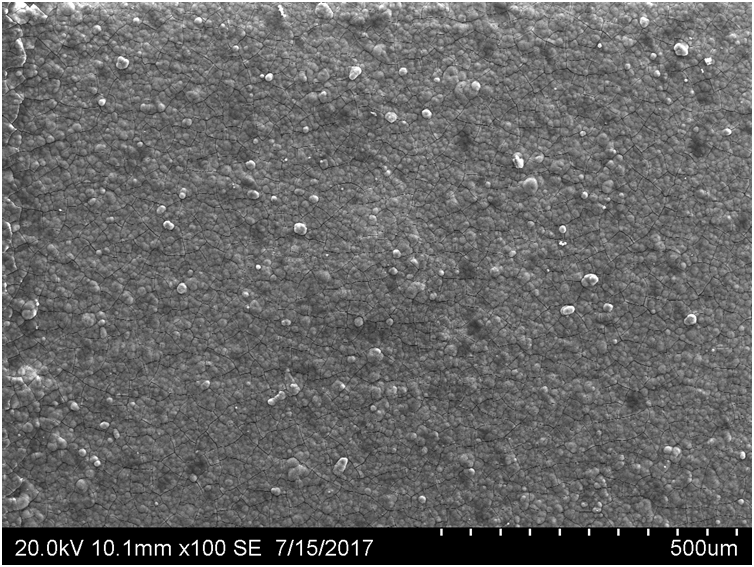

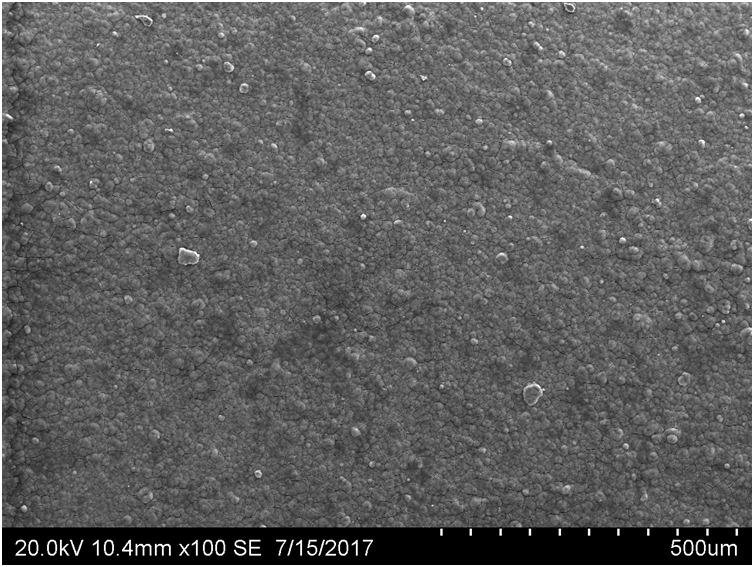


**100 μm**

**(b)**


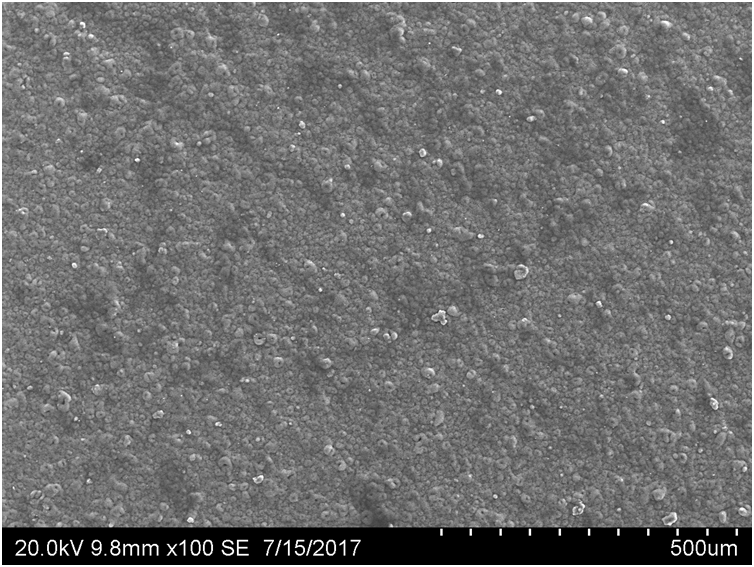

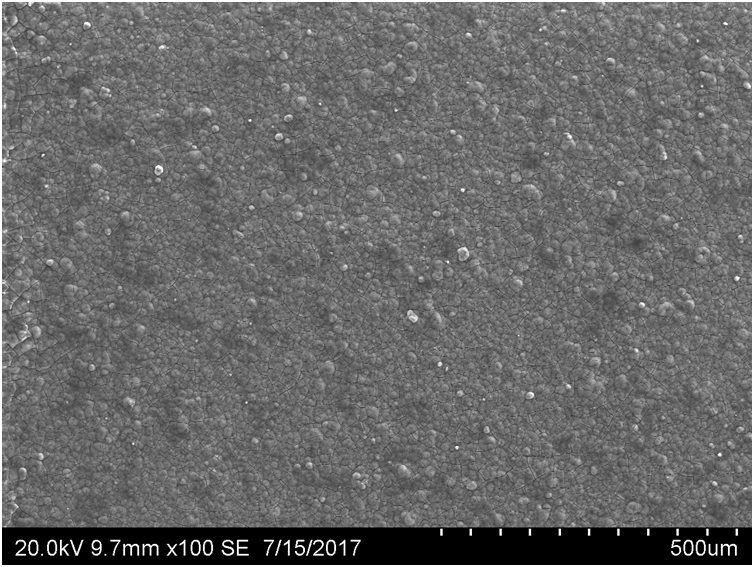

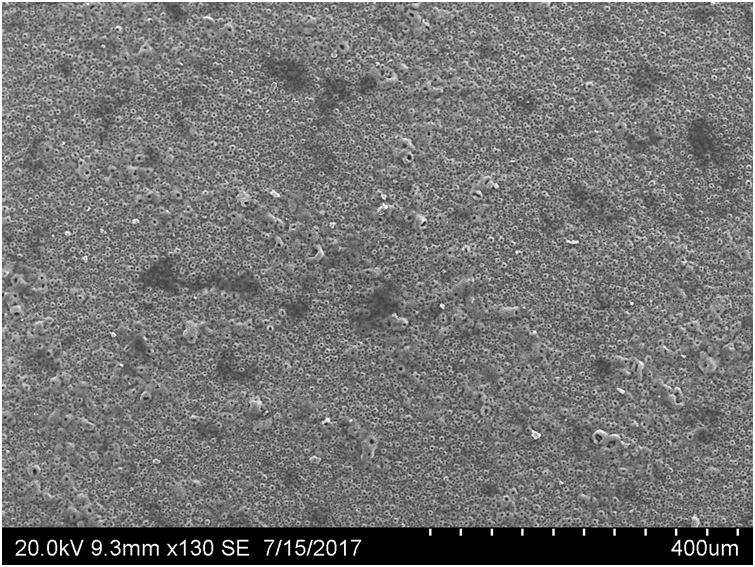

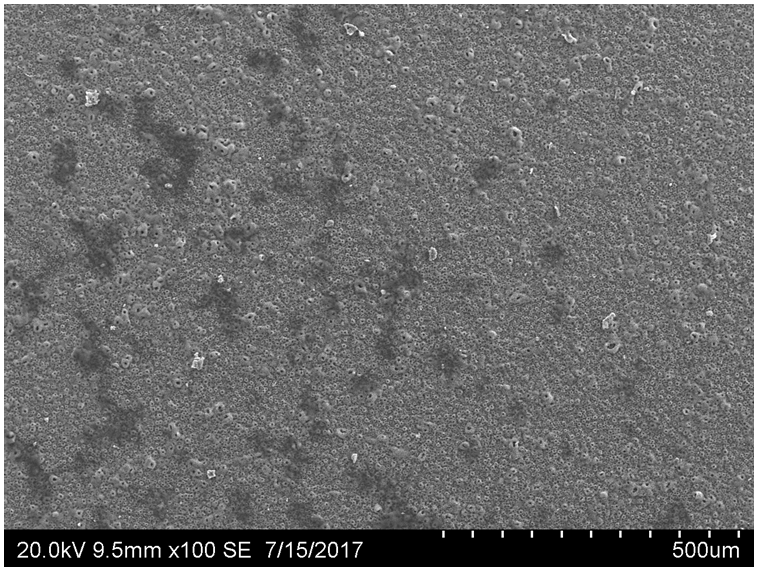


**100 μm**

**(c)**

**1% 2h**

**1% 4h**

**1% 8h**

**1% 16h**

**2% 2h**

**2% 4h**

**2% 8h**

**2% 16h**

**5% 2h**

**5% 4h**

**5% 8h**

**5% 16h**

**100 μm**

**100 μm**

**100 μm**

**100 μm**

**100 μm**

**100 μm**

**100 μm**

**100 μm**

**100 μm**

**Apatite film**

**Apatite film**

**Apatite film**

**Apatite film**

**Apatite film**

**Apatite film**

**Apatite film**

**Apatite film**

**Gel area**

**Gel area**

**Gel area**

**Gel area**

Figure S1 Apatite-inducing ability of the chemical grafted samples treated with different concentrations of glutaraldehyde: (a) 1%, (b) 2%, and (c) 5%.


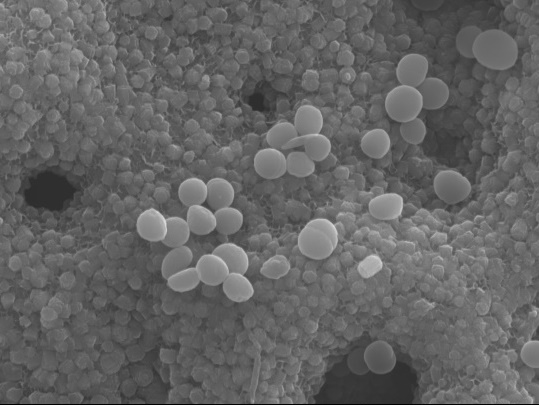

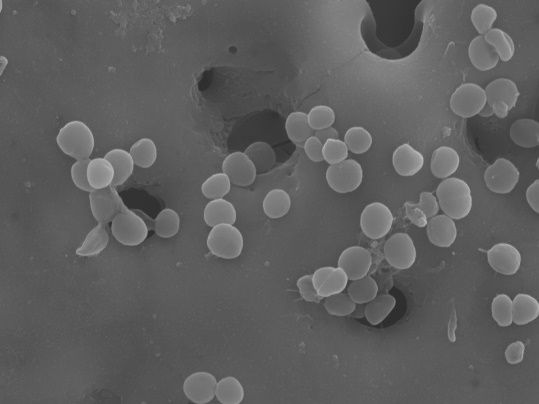

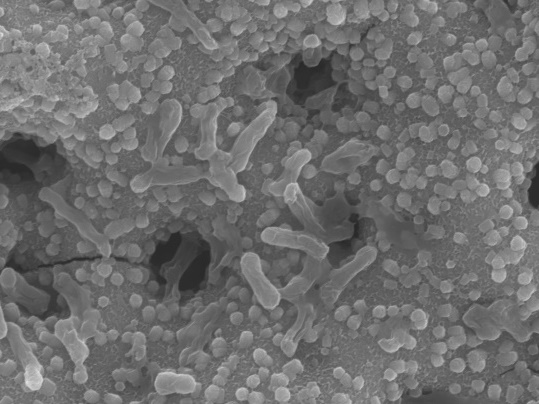

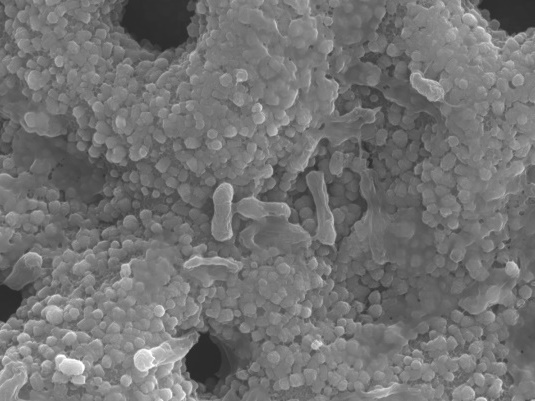

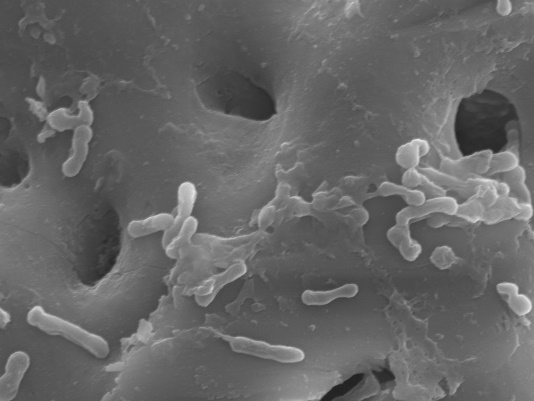

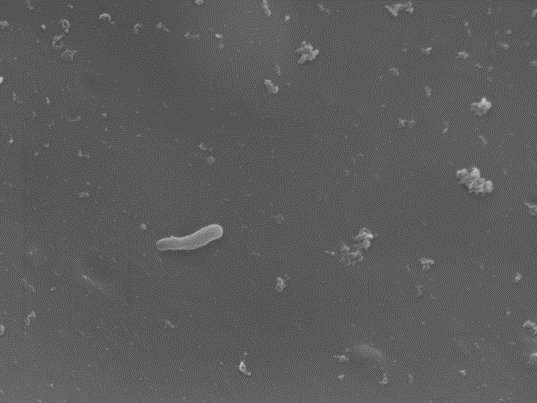

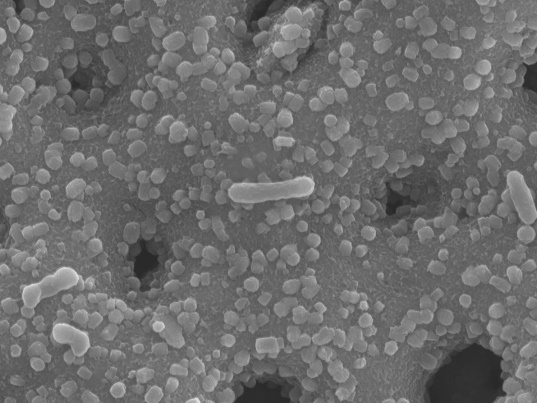


**2 μm**

**2 μm**

**2 μm**

**2 μm**

**2 μm**


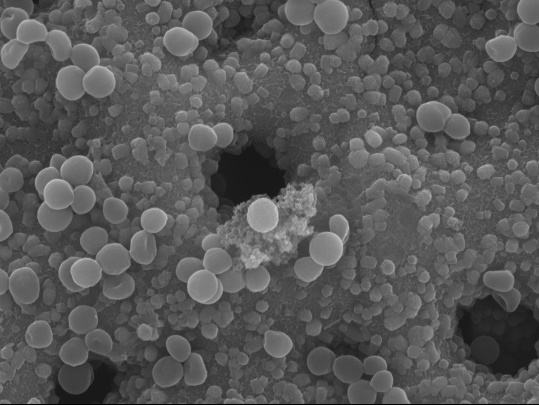


**2 μm**

**2 μm**

**2 μm**


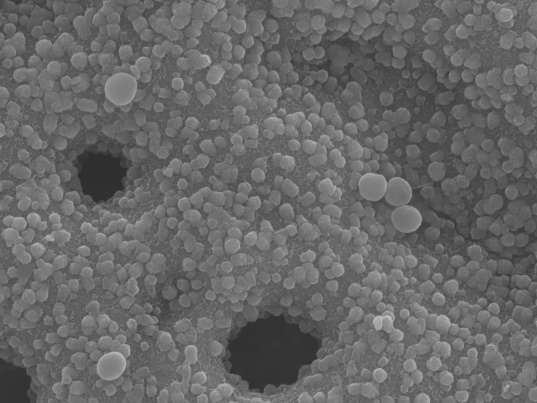

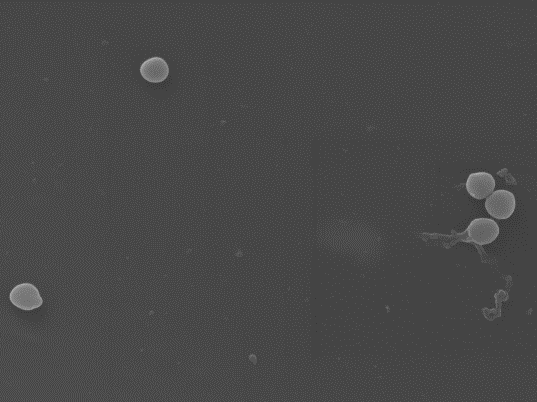


**2 μm**

**2 μm**

**MAO**

**MAO**

**HT**

**HT**

**CG**

**CG**

**UV-CS loaded area**

**UV-CS unloaded area**

**UV-CS loaded area**

**UV-CS unloaded area**

**(a)**

**(b)**

***E. Coli***

***E. Coli***

***E. Coli***

***E. Coli***

***E. Coli***

***S. aureus***

***S. aureus***

***S. aureus***

***S. aureus***

***S. aureus***

Figure S2 SEM morphologies of the bacteria incubated samples with different surface features for 1 day: (a) *E. coli* group, and (b) *S. aureus* group.


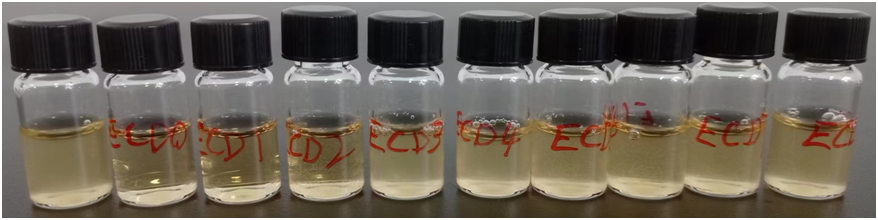

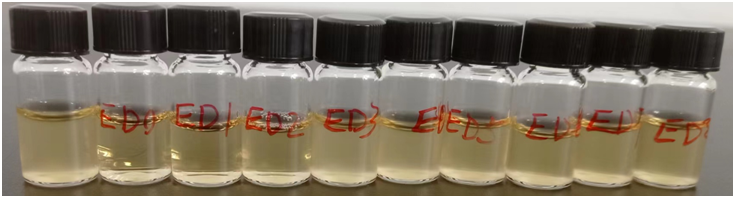

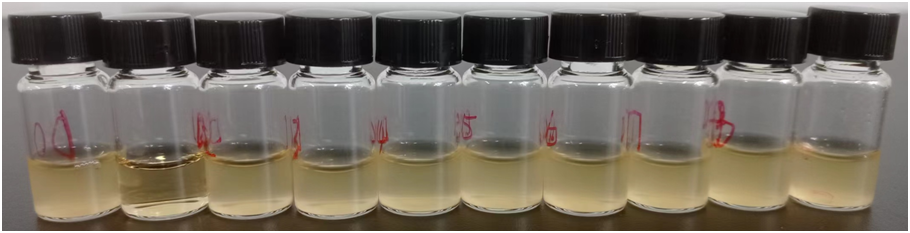

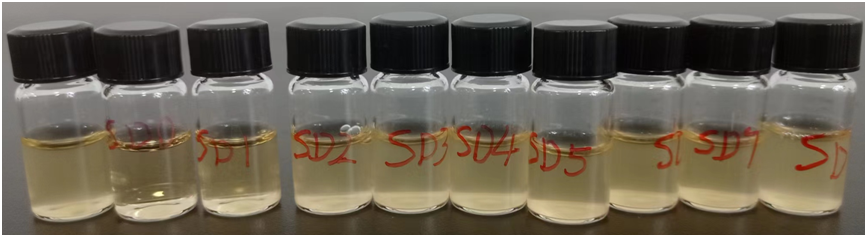


***S. aureus***

***E. coli***

**Ciprofloxacin**

**Ciprofloxacin loaded UV-CS**

**Ciprofloxacin**

**Ciprofloxacin loaded UV-CS**

Figure S3 Images for the bacteria inoculated nutrient broth with different concentrations of antibacterial agents.
